# Environmental DNA metabarcoding of cow dung reveals taxonomic and functional diversity of invertebrate assemblages

**DOI:** 10.1111/mec.15734

**Published:** 2020-12-15

**Authors:** Eva Egelyng Sigsgaard, Kent Olsen, Morten D. D. Hansen, Oskar Liset Pryds Hansen, Toke Thomas Høye, Jens‐Christian Svenning, Philip Francis Thomsen

**Affiliations:** ^1^ Department of Biology Aarhus University Aarhus Denmark; ^2^ Natural History Museum Aarhus Aarhus Denmark; ^3^ Department of Bioscience Aarhus University Rønde Denmark

**Keywords:** conservation biology, environmental DNA, insects, invertebrates

## Abstract

Insects and other terrestrial invertebrates are declining in species richness and abundance. This includes the invertebrates associated with herbivore dung, which have been negatively affected by grazing abandonment and the progressive loss of large herbivores since the Late Pleistocene. Importantly, traditional monitoring of these invertebrates is time‐consuming and requires considerable taxonomic expertise, which is becoming increasingly scarce. In this study, we investigated the potential of environmental DNA (eDNA) metabarcoding of cow dung samples for biomonitoring of dung‐associated invertebrates. From eight cowpats we recovered eDNA from 12 orders, 29 families, and at least 54 species of invertebrates (mostly insects), representing several functional groups. Furthermore, species compositions differed between the three sampled habitats of dry grassland, meadow, and forest. These differences were in accordance with the species’ ecology; for instance, several species known to be associated with humid conditions or lower temperatures were found only in the forest habitat. We discuss potential caveats of the method, as well as directions for future study and perspectives for implementation in research and monitoring.

## 1 INTRODUCTION

Studies demonstrating significant reductions in insect biomass are currently an issue of scientific debate and public concern (Hallmann et al., 2017; Seibold et al., 2019; Wagner, 2020), with many terrestrial invertebrate species showing large declines in abundance (Dirzo et al., 2014). Herbivore dung supports a rich biodiversity of such invertebrates in terrestrial habitats (Floate, 2011; Lee & Wall, 2006; Skidmore, 1991), which in turn serve as an important food source for insectivorous birds and mammals (Liu et al., 2019; Skidmore, 1991; Vickery et al., 2001; Virgós et al., 2004). More than 400 species of insects are known to be associated with dung in Britain alone (Skidmore, 1991), and additional groups such as mites (Arjomandi et al., 2013), centipedes (Wall & Strong, 1987), nematodes (Weller et al., 2010) and fungi (Richardson, 2001) are also numerous in these miniature ecosystems. Dung provides a vital food source as well as protection and moisture for its inhabitants. Several insect species feed on the dung itself (e.g., Scarabaeidae, Sepsidae etc), while other species feed on dung‐associated fauna as predators (e.g., Staphylinidae, Hydrophilidae, Muscidae etc) or on dung‐associated fungal spores (e.g., Ptillidae, Acari, Collembola) (Skidmore, 1991). The decline in dung‐associated fauna has been most thoroughly illustrated using dung beetles as indicators (Aguilar‐Amuchastegui & Henebry, 2007; Davis et al., 2001; Filgueiras et al., 2015). Dung beetle species richness is positively associated with grazing continuity ‐ especially for habitat specialists (Buse et al., 2015), while grazing abandonment and hunting of medium‐ and large‐bodied mammals have been shown to lead to significant decreases in alpha diversity and biomass / abundance of dung beetles (Nichols et al., 2009; Tonelli et al., 2018). In European ecosystems, dung beetles were formerly abundant and diverse, but especially large‐bodied species have declined in association with the progressive loss of megafauna since the Late Pleistocene (Sandom et al., 2014; Schweiger & Svenning, 2018). Thus, if appropriately tailored to the ecosystem and its history (Schweiger et al., 2019), trophic rewilding (Svenning et al., 2016) via restoring megafauna is expected to benefit dung‐beetle faunas (Brompton, 2018) by expanding the ecospace (increasing the amount and diversity of organic matter; Brunbjerg et al., 2017).

Studies of dung‐fauna communities have traditionally relied on methods such as dissolving cowpats in water, or using anoxic conditions or funnels to extract the animals (Skidmore, 1991). These methods are needless to say both messy and cumbersome, and will probably overlook species that are only in brief contact with the cowpats, such as flying species that only feed on dung as adults. Moreover, considerable taxonomic expertise is required to be able to morphologically identify all dung‐associated taxa. This expertise is generally declining (Hopkins & Freckleton, 2002; Sangster & Luksenburg, 2015; Wheeler et al., 2004) and even for experts, morphology‐based taxonomic identification of arthropods poses difficulties, e.g., for species with large intraspecific morphological variation, closely related species, and juvenile stages. With insects in steep decline globally, more intensive data collection is needed (Montgomery et al., 2020), but this will require time‐ and cost‐efficient monitoring approaches.

Within the last decade, it has been demonstrated that various sources of contemporary environmental samples contain DNA from a diverse range of macroorganisms, and that such noninvasive environmental DNA (eDNA) analyses have the potential to supplement many traditional sampling approaches in ecology (Sigsgaard, Jensen, et al., 2020; Taberlet et al., 2018; Thomsen & Willerslev, 2015). Environmental DNA analyses of soil (Taberlet, Prud'Homme, et al., 2012; Yoccoz et al., 2012; Zinger et al., 2018) and water (Ficetola et al., 2008; Sigsgaard et al., 2016; Stat et al., 2017; Thomsen, Kielgast, Iversen, Møller, et al., 2012; Thomsen, Kielgast, Iversen, Wiuf, et al., 2012) have proven successful, although many other sources of eDNA have also provided valuable data. For example, insect eDNA has been found on wild flowers (Thomsen & Sigsgaard, 2019), pollen attached to insects has been used for retrieving information on plant–pollinator interactions (Bell et al., 2017; Pornon et al., 2017) and eDNA from bulk samples (insect soups) reflect the species present in the samples (Arribas et al., 2018; Elbrecht et al., 2016; Yu et al., 2012). Furthermore, eDNA analyses of faecal samples have been especially popular for obtaining insights on animal diet for e.g., mammals (Berry et al., 2017; Pompanon et al., 2012; Valentini, Miquel, et al., 2009; Valentini, Pompanon, et al., 2009), fish (Jensen et al., 2018), birds (Thalinger et al., 2016) and insects (Valentini, Miquel, et al., 2009; Valentini, Pompanon, et al., 2009). DNA metabarcoding—high‐throughput sequencing of PCR amplicons using generic primers—is currently the most efficient approach for analysing eDNA samples (Taberlet, Coissac, et al., 2012; Taberlet et al., 2018; Zinger et al., 2019), and was used in most of the above‐mentioned studies.

Here, we analyse eDNA from cattle dung using eDNA metabarcoding, but with a focus on the dung‐associated invertebrate fauna rather than the diet of the herbivore. Van der Heyde et al. (2020) recently found beetle DNA in scat samples from herbivores, supporting the idea that faecal samples may contain eDNA from invertebrates that have come in contact with the substrate after defecation. We hypothesize that dung‐associated invertebrates leave DNA traces in the dung, and investigate: (i) to what extent this eDNA can be obtained, (ii) whether it reflects the taxonomic and functional diversity of the local assemblage of dung‐associated fauna; and (iii) whether the assemblage detected in dung from a forest is different to that found in dung from two open grassland habitats.

## 2 MATERIALS AND METHODS

### 2.1 Study sites

**FIGURE 1:**
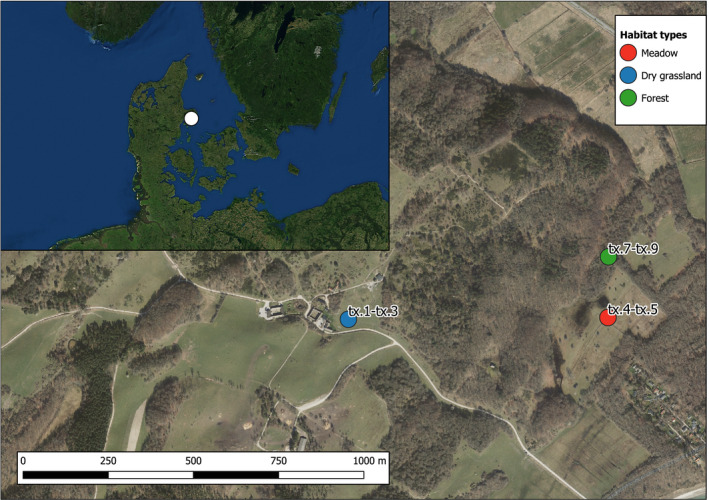
Map of sampling sites in Mols Bjerge with inset overview map of Denmark. Sample names are indicated for each site. Map data: (main map) Agency for Data Supply and Efficiency. GeoDanmark Ortofoto. 2019, (inset map) ESRI. World Imagery 2013

The study was carried out in the Natura 2000 site Mols Bjerge, located in Mols Bjerge National Park in Denmark (56°13′36″N, 10°34′33″E; Figure 1). Within this area, the Natural History Museum Aarhus owns a highly biodiverse natural hotspot of 1.5 km^2^ managed with conservation of biodiversity and ecosystem restoration as its primary purpose. The main habitat types are dry grassland, deciduous forest, and rich fen/meadows. The area is known as Rewilding Mols at The Mols Laboratory, where a mixed population of feral Galloway cattle (*Bos taurus* Linnaeus, 1758) and Exmoor ponies (*Equus caballus* Linnaeus, 1758) have been introduced in accordance with the idea of trophic rewilding (Svenning et al., 2016) and the population densities are regulated by humans based on resource availability and animal condition, without any supplementary feeding (micronutrients and water are provided). The Rewilding Mols project was launched in November 2016 with late autumn population densities of the two large herbivore species counting 13 and 12 in 2016, 22 and 18 in 2017, 32 and 27 in 2018, and 44 and 36 in 2019, respectively. At the time of sampling in June 2019, 43 cattle and 30 horses were present.

### 2.2 Sampling

Dung samples from the cattle were collected in the Rewilding Mols project area in June 2019 on the following dates; tx.1–tx.3: 12 June at 1:35–1:55 p.m., tx.4–tx.6: 14 June at 4:34–4:40 p.m., and tx.7–tx.9: 17 June at 4:57–5:07 p.m. (Figure S1). Based on the visual characteristics of their surface, we selected the nine individual cowpats so that they appeared to have the same relative age (Figure S1). One dung sample of ca. 5 ml was collected from each of the nine individual cowpats found in three different habitats; dry grassland (tx.1–tx.3), meadow (tx.4–tx.6), and forest (tx.7–tx.9; Figure 1, Figure S1). Each dung sample consisted of five subsamples from the same cowpat, each of ca. 1 ml, which was pooled together in a sterile 5 ml Eppendorf tube using single‐use nitrile gloves, disposable face mask and plastic spoons. During collection, samples were thoroughly inspected to ensure that they did not contain any visible animals. All samples were kept in a box with ice blocks immediately after sampling and stored at –20°C after return from the field (maximum a few hours after sampling). They were kept at –20°C until DNA extraction.

### 2.3 DNA extraction

DNA extractions were performed in the clean laboratory facility at the Department of Biology, Aarhus University, which is a dedicated laboratory for working with samples of low DNA concentration. Regular decontamination routines are in place, including UV light, and only pre‐PCR work is carried out in this laboratory. DNA was extracted using QIAamp Fast DNA Stool Mini Kit (Qiagen, Germantown, USA). Before extraction, the samples were transferred to 50 ml falcon tubes, using the handle of a metal spoon, to allow thorough mixing of the dung. Spoons were cleaned before use and between samples by wiping twice with DNAaway, then wiping with ethanol, and lastly leaving the spoons under UV light for 10 min with the front surface of the spoon facing upwards, and 10 min with the back surface facing upwards. Despite visual inspection during sampling, two unidentified larvae were found in the sample tx.5, and were removed before sample mixing. Samples were mixed thoroughly by vortexing, and a subsample of ~220 mg was taken out from each sample for extraction. The manufacturer's protocol for human DNA analysis was thereafter followed with the following exceptions; after addition of InhibitEx buffer and vortexing, samples were shaken on a thermomixer for 2 h and were then centrifuged for 5 min. Elution of DNA was done in 2*60 µl ATE buffer, with an incubation of 5 min at room temperature before each centrifugation. An extraction blank was included throughout the extraction process, and final DNA extracts were stored at –20°C.

### 2.4 PCR amplification

For DNA metabarcoding, we used a primer set (BF1 and BR1) targeting the mitochondrial cytochrome c oxidase subunit I (COI) gene and designed for invertebrates (Elbrecht & Leese, 2017). Primers were uniquely tagged. Tags were designed using the OligoTag program (Coissac, 2012), and consisted of six nucleotides with a distance of at least three bases between any two tags. Tags were preceded by two or three random bases; NNN or NN (De Barba et al., 2014) to increase sequence complexity, and identical tags were used on the forward and reverse primers for each sample to avoid tag jumps (Schnell et al., 2015).

Two replicate PCR reactions were carried out for each sample including the extraction blank, using identical tags for PCR replicates, but a unique tag for each sample. Two PCR blanks were also included. PCR reactions were performed in 25 µl volumes of 3 µl template DNA, 10 ul HotStarTaq Master Mix (Qiagen), 8 µl ddH_2_O, 1.5 µl of each primer (10 µM), and 1 µl bovine serum albumin (BSA; 20 mg/ml). Thermocycling parameters were: 95°C for 15 min, 50 cycles of 94°C for 30 s, 46°C for 30 s, and 72°C for 1 min, and a final elongation of 72°C for 7 min. The initial heat deactivation, denaturation and extension steps were chosen based on the guidelines for the HotStarTaq Master Mix, while the annealing temperature followed Elbrecht and Leese (2015). The number of PCR cycles was determined from previous eDNA optimization experiments in the laboratory with the same primers (unpublished data).

Fragment sizes were verified on 2% agarose gel stained with GelRed. PCR products were mixed in two pools each containing one PCR replicate of each sample, one replicate of the extraction control and one PCR blank (2 µl per replicate). In addition to the samples included in this study, 17 other samples were included in the pools, also with one PCR replicate in each pool. The pools were purified using Qiagen's MinElute PCR purification kit, along with a purification blank. The manufacturer's protocol was followed with the exception that samples were incubated with the elution buffer (2*20 µl EB) over two rounds of 37°C for 10 min.

### 2.5 Library building and next‐generation sequencing

Approximately 750 ng of PCR product from each pool, as determined with the Qubit HS DNA Kit (Thermofisher Scientific, Carlsbad, CA, USA), was used as input for the libraries. Library building was performed on the purification blank and each of the purified pools of PCR products using the TruSeq DNA PCR‐free LT Sample Prep kit (Illumina, San Diego, California). The concentration and fragment size distribution of the libraries were verified using Qubit and an Agilent 4200 TapeStation before sequencing (150 bp paired‐end) in a single run on the Illumina MiSeq platform, at the Microbiology Section, Department of Biology, Aarhus University.

### 2.6 High‐throughput sequencing data analyses

After primer removal and demultiplexing using the software Cutadapt (Martin, 2011), Illumina sequences were trimmed with Sickle (Joshi & Fass, 2011), applying a required average quality score of 28 in the sliding window. The sequences were then analysed using DADA2 (Callahan et al., 2016), to clean the data from errors generated during PCR and sequencing (Ficetola et al., 2015; Murray et al., 2015; Olds et al., 2016). The error filtering in DADA2 is based on error models inferred from the data itself, and was therefore done separately for each of the four fastq files (reads 1 and 2 for each of the two libraries). Initial filtering was set to allow a maximum of two expected errors (maxEE = 2) and to truncate reads at the first instance of a quality score at or below 2 (truncQ = 2, default). Forward and reverse reads were then merged (minimum of 5 bp overlap following Frøslev et al., 2017, no mismatches allowed) and likely chimeras were removed with the DADA2 function removeBimeraDenovo. All remaining sequences were then searched against the GenBank nt database (Benson et al., 2005) on 17 October 2019, using blastn (Altschul et al., 1990), requesting a maximum of 500 aligned sequences per query, and minimum thresholds of 90% query coverage per high‐scoring segment pair and 80% sequence similarity. The blast hits displaying an incomplete final coverage of the query sequence were removed and taxonomically classified using the R package taxize (Chamberlain & Szocs, 2013). Sequences classified as metazoan were then searched against the Barcode of Life Data Systems (BOLD; Ratnasingham & Hebert, 2007) and taxonomically classified using the bold package (Chamberlain, 2019) in r version 3.5.0 (R Core Team, 2019). Each sequence was then assigned to the lowest common ancestor of all matching taxa that overlapped in their range of sequence similarities with that found for the taxon (or taxa) with the highest sequence similarity; i.e., if the best hit was for example a 99% match to a certain fly species, but other BOLD sequences from this species yielded only a 98.5% match, all taxa with a hit of at least 98.5% were considered. If there was no overlap in sequence similarity between the taxon producing the best hit, and other taxa, and this highest‐matching taxon produced a hit of at least 98% similarity, the sequence was assigned to species. For assignment to genus or family level, thresholds of 91% and 83% sequence similarity were used, based on calculations following Alberdi et al. (2018). Until this point, data analyses were conducted using the high‐performance computing facility GenomeDK, Center for Genome Analysis and Personalized Medicine, Aarhus University, while the following analyses were conducted on a local computer. To produce a conservative estimate of the diversity obtained by eDNA, we excluded taxa found in only a single PCR replicate across all samples (Alberdi et al., 2018; Thomsen & Sigsgaard, 2019), and we report this as the final data (Table 1). Sequences identified as originating from cow (*Bos taurus*) were also removed from the final data.

**TABLE 1:** List of metazoan taxa detected with eDNA analysis of cow dung samples

Class	Order	Family	Final identification	Dung‐associated	Grassland	Meadow	Forest	No. of reads
Arachnida	Mesostigmata	Macrochelidae	*Macrocheles*sp.	Indirectly				18,027
Arachnida	Sarcoptiformes	Ceratozetidae	Trichoribates incisellus	Indirectly		x	x	158
Arachnida	Sarcoptiformes	Chamobatidae	Chamobates birulai	Indirectly			x	313
Arachnida	Sarcoptiformes	NA	*Sarcoptiformes*sp.	–				16
Collembola	Entomobryomorpha	Entomobryidae	*Entomobrya* sp.	Indirectly				73
Collembola	Entomobryomorpha	Isotomidae	Desoria grisea	Indirectly			x	409
Collembola	Entomobryomorpha	Isotomidae	Isotomurus fucicolus	Indirectly			x	6
Collembola	Poduromorpha	Hypogastruridae	Ceratophysella denticulata	Indirectly			x	922
Collembola	Poduromorpha	Hypogastruridae	Hypogastrura assimilis	Indirectly	x	x	x	6601
Collembola	Symphypleona	Sminthuridae	Sminthurus viridis	Indirectly	x			79
Insecta	Coleoptera	Curculionidae	Hypera plantaginis	No	x	x	x	50
Insecta	Coleoptera	Histeridae	Margarinotus ventralis	Yes	x	x		982
Insecta	Coleoptera	Hydrophilidae	Cercyon haemorrhoidalis	Yes	x			211
Insecta	Coleoptera	Hydrophilidae	Cercyon pygmaeus	Yes	x			218
Insecta	Coleoptera	Hydrophilidae	Cercyon quisquilius	Yes	x			188
Insecta	Coleoptera	Hydrophilidae	Sphaeridium bipustulatum	Yes	x			96
Insecta	Coleoptera	Hydrophilidae	Sphaeridium lunatum	Yes	x	x		6172
Insecta	Coleoptera	Hydrophilidae	Sphaeridium scarabaeoides	Yes	x	x		595
Insecta	Coleoptera	Phalacridae	*Olibrus*sp.	No				5842
Insecta	Coleoptera	Scarabaeidae	Aphodius depressus	Yes			x	432
Insecta	Coleoptera	Scarabaeidae	Aphodius haemorrhoidalis	Yes	x	x		4706
Insecta	Coleoptera	Scarabaeidae	Aphodius sphacelatus	Yes		x		21
Insecta	Coleoptera	Scarabaeidae	Aphodius sticticus	Yes			x	17
Insecta	Coleoptera	Staphylinidae	Oxytelus laqueatus	Yes			x	8976
Insecta	Dermaptera	Forficulidae	Forficula auricularia	Indirectly	x			16
Insecta	Diptera	Anisopodidae	*Sylvicola*sp.	Yes				1867
Insecta	Diptera	Anthomyiidae	Hylemya vagans	Yes			x	1139
Insecta	Diptera	Chironomidae	*Smittia*sp.	Yes				178
Insecta	Diptera	Muscidae	Azelia nebulosa	Yes			x	268
Insecta	Diptera	Muscidae	Haematobia irritans	Yes	x	x		550
Insecta	Diptera	Muscidae	Hebecnema umbratica	Yes		x	x	1423
Insecta	Diptera	Muscidae	Mesembrina meridiana	Yes		x		10
Insecta	Diptera	Muscidae	Musca autumnalis	Yes	x	x		102,361
Insecta	Diptera	Muscidae	Mydaea urbana	Yes			x	213
Insecta	Diptera	Muscidae	Neomyia cornicina	Yes	x	x		30,027
Insecta	Diptera	NA	*Diptera*sp.	–				14
Insecta	Diptera	Psychodidae	Psychoda grisescens	Yes			x	147
Insecta	Diptera	Psychodidae	Psychoda phalaenoides	Yes		x	x	239,498
Insecta	Diptera	Psychodidae	Psychoda setigera	Yes	x		x	6158
Insecta	Diptera	Psychodidae	Psychoda trinodulosa	Yes	x	x	x	17,653
Insecta	Diptera	Scathophagidae	Scathophaga stercoraria	Yes	x	x		2812
Insecta	Diptera	Sepsidae	Saltella sphondylii	Yes	x	x		23,025
Insecta	Diptera	Sepsidae	*Sepsis*sp.	Yes				11,867
Insecta	Diptera	Sepsidae	Sepsis cynipsea	Yes	x	x		86,068
Insecta	Diptera	Sepsidae	Sepsis duplicata	Yes	x	x		2058
Insecta	Diptera	Sepsidae	Sepsis thoracica	Yes	x	x		1153
Insecta	Diptera	Sphaeroceridae	Coproica lugubris	Yes	x	x		802
Insecta	Diptera	Sphaeroceridae	Norrbomia sordida	Yes		x		77
Insecta	Diptera	Sphaeroceridae	Lotophila atra	Yes	x	x	x	7885
Insecta	Diptera	Sphaeroceridae	Spelobia clunipes	Yes	x	x	x	58,006
Insecta	Diptera	Stratiomyidae	Microchrysa polita	Yes	x			1869
Insecta	Hemiptera	Aphididae	Euceraphis betulae	No			x	11
Insecta	Hemiptera	Miridae	Rhabdomiris striatellus	No	x			55
Insecta	Hemiptera	Pentatomidae	*Dolycoris*sp.	No				79
Insecta	Hymenoptera	Formicidae	Lasius niger	No	x			24
Insecta	Phthiraptera	Bovicoliidae	Bovicola bovis	Cow parasite	x			38
Chromadorea	Strongylida	Ancylostomatidae	Bunostomum phlebotomum	Cow parasite		x	x	29

### 2.7 Rarefaction analyses

To check whether sequencing depth was sufficient to capture the taxonomic diversity represented in the PCR replicates, rarefaction curves for each of the individual replicates were performed using the function rarecurve in the R package vegan v. 2.4‐6 (Oksanen et al., 2018).

### 2.8 Accumulation analyses

To determine whether sampling effort had been sufficient to cover taxonomic diversity within each habitat, and within the entire study area, respectively, taxon accumulation curves were performed using the function specaccum from vegan. The “exact” species accumulation method was used, which finds the mean species richness across sites.

### 2.9 Differentiation analyses

In order to investigate whether the invertebrate assemblages were differentiated according to habitat, we performed several different analyses. Raup–Crick distances, which are presence‐absence based, were calculated with the vegdist function in vegan, and subjected to a permutational analysis of variance (PERMANOVA) test using the function adonis (number of permutations = 999). Because the dissimilarity data did not meet the assumption of multivariate homogeneity of group dispersions (permutation test, *p* < .05), the data were transformed using inverse normal transformation. Using the package pheatmap (Kolde, 2019), we then produced heatmaps combined with hierarchical clusters, showing the presence or absence of taxa in specific samples, the similarity in assemblage composition between different samples, and the similarity between taxa with regard to the samples they appeared in. The same was done for trophic groups, as modified from Skidmore (1991). Clustering was set to the average‐linkage method and was done using the Raup–Crick distances from vegdist, transformed with cube transformation (n^1/3^) to obtain an appropriate scale for the figure. Additionally, a detrended correspondence analysis (DCA) of invertebrate assemblages in the different samples was performed, using the decorana function in vegan (Hill & Gauch, 1980; Oksanen & Minchin, 1997). The read abundances were not transformed for this analysis, as they did not have any effect on the grouping of the samples. All analyses were performed in r v. 3.6.1 (R Core Team, 2019).

## 3 RESULTS

### 3.1 PCR amplification

One sample (tx.6) from the meadow habitat (Figure S1) did not yield visible gel bands, and was therefore not included in sequencing libraries. All other PCRs on the eight remaining samples gave visible bands and were included in sequencing libraries along with the DNA extraction blank and PCR blanks (the latter two gave no visible bands, but were sequenced nonetheless).

### 3.2 DNA metabarcoding reads

A total of 15,336,282 raw reads corresponding to 7,668,141‬ read pairs were produced on the Illumina MiSeq platform. We obtained similar sequencing depth across the two libraries (PCR replicates) with 4,514,166 and 3,153,975 read pairs obtained per library, respectively. After initial data cleaning and merging of paired reads, a total of 1,453,478 reads were retained in total for the samples included in this study (excluding blanks), of which 652,590 reads were classified as metazoans and appeared in at least two PCR replicates in the data set. The eight samples tx.1–tx.9 (excl. tx.6) had similar sequence depths with 81,574 ± 19,422 final reads (mean ± *SEM*). No metazoan sequences were retained in the extraction blank or PCR blanks.

**FIGURE 2:**
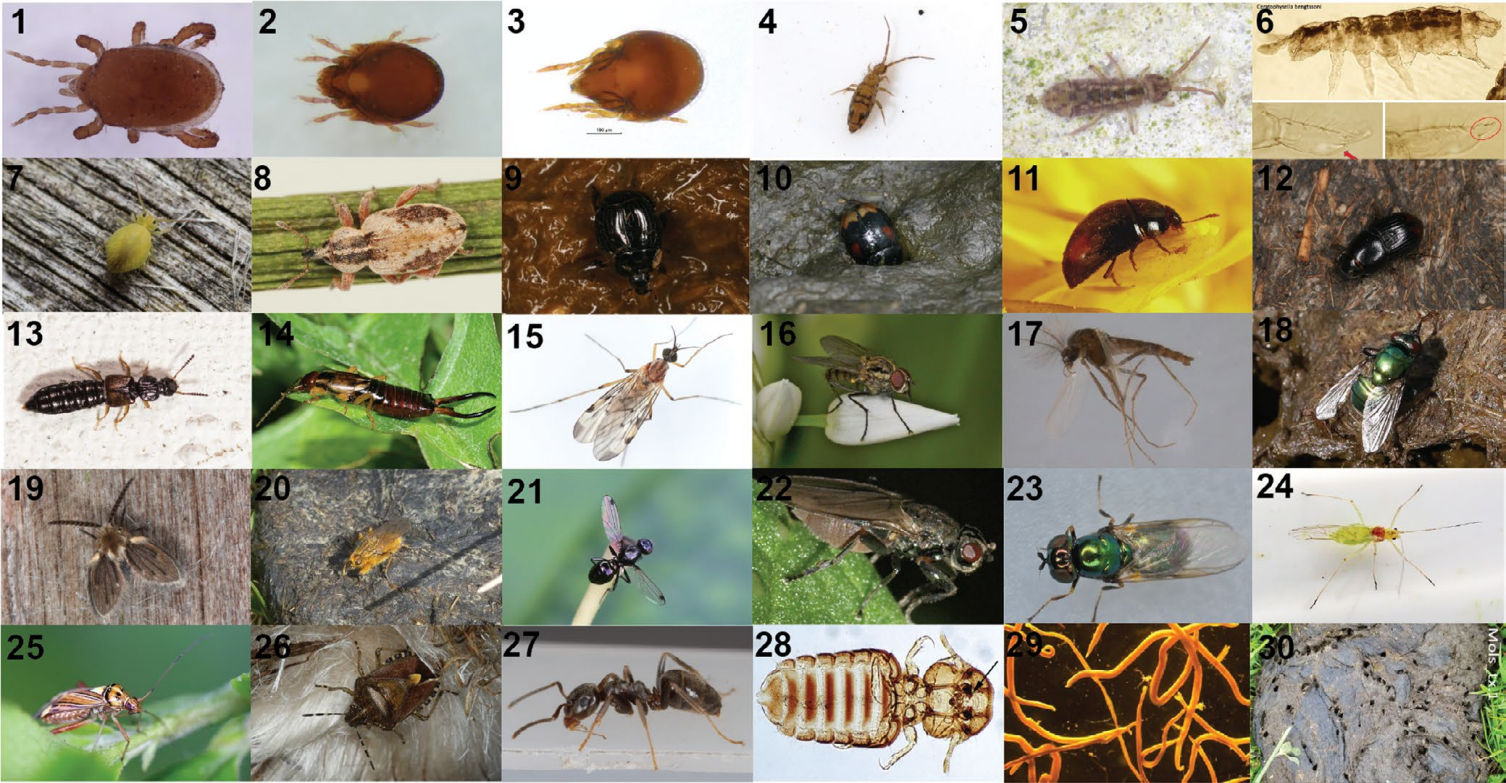
Photos of invertebrate families found with eDNA in dung samples in this study. A representative for each family is shown. *The taxon found in the study is different from the one in the example photograph, see Table 1. See the Acknowledgements section for photograph credits. ARACHNIDA: (1) Macrochelidae (Macrocheles montanus*); (2) Ceratozetidae (*Trichoribates incisellus*); (3) Chamobatidae (*Chamobates birulai*); COLLEMBOLA; (4) Entomobryidae (Entomobrya nivalis*); (5) Isotomidae (Isotomurus maculatus*); (6) Hypogastruridae (Ceratophysella bengtssoni*); (7) Sminthuridae (*Sminthurus viridis*); INSECTA: (8) Curculionidae (*Hypera plantaginis*); (9) Histeridae (Margarinotus neglectus*); (10) Hydrophilidae (*Sphaeridium lunatum*); (11) Phalacridae (*Olibrus bicolor*); (12) Scarabaeidae (*Aphodius haemorrhoidalis*); (13) Staphylinidae (*Oxytelus laqueatus*); (14) Forficulidae (*Forficula auricularia*); (15) Anisopodidae (*Sylvicola* sp.); (16) Anthomyiidae (*Hylemya vagans*); (17) Chironomidae (Smittia nudipennis*); (18) Muscidae (*Neomyia cornicina*); (19) Psychodidae (*Psychoda* sp.); (20) Scathophagidae (*Scathophaga stercoraria*); (21) Sepsidae (*Sepsis* sp.); (22) Sphaeroceridae (*Lotophila atra*); (23) Stratiomyidae (*Microchrysa polita*); (24) Aphididae (Euceraphis betulae*); (25) Miridae (*Rhabdomiris striatellus*); (26) Pentatomidae (Dolycoris baccarum*); (27) Formicidae (*Lasius niger*); (28) Bovicoliidae (*Bovicola* sp.*); CHROMADOREA: (29) Ancylostomatidae (*Bunostomum* sp.*). Panel (30) shows one of the sampled cowpats, Mols_tx.1

**FIGURE 3:**
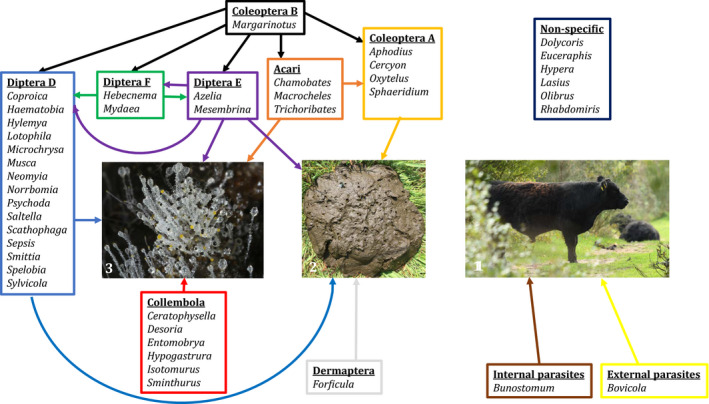
Trophic network representation of invertebrate genera found in the study. Modified from Skidmore (1991). (i) Cow from the sampling site; (ii) cow dung; (iii) fungus on cow dung. Coleoptera A, beetles and their larvae which feed entirely or mainly on the dung itself (some of these are probably also partly feeding on other arthropods, fungi and bacteria); Coleoptera B, predatory beetles and their larvae which feed on other arthropods; Diptera D, flies and mosquitos whose larvae feed on the dung itself and associated fungi and bacteria; Diptera E, members of Muscidae in which the larvae feed as Diptera D in the first instars but become facultative carnivores in the final instar; Diptera F, members of Muscidae in which the larvae are obligate carnivores; Collembola, springtails, which are hexapods often numerous in dung where they feed on associated fungi, though most belong to the soil fauna; Acari, mites, which are arachnids often numerous in dung and feed on dung and associated fungi (Trichoribates, Chamobates) or sometimes as predators or parasites on insect larvae and eggs (Macrocheles); Dermaptera, earwigs, which are insects not primarily associated with dung, but which can utilise dungpats for laying eggs and brooding the nymphs; Nonspecific insects found in the study, which are not associated with dung but are abundant at the site. See also Discussion section. Photo credits: Ole Martin (left), Philip Francis Thomsen (middle), Morten D. D. Hansen (right)

The final sequences represented 64 amplicon sequence variants (ASVs, see e.g., Callahan et al., 2017) covering 12 orders, 29 families and at least 54 different species of invertebrates (of which seven are only identified to genus level and two to order level; Table 1, Figures 2 and 3). The vast majority of species obtained were insects, and the order with most sequencing reads was Diptera, which accounted for the top seven most abundant species (Table 1). The top three most abundant taxa (*Psychoda phalaenoides* Linnaeus, 1758, *Musca autumnalis* De Geer, 1776, and *Sepsis cynipsea* Melander and Spuler, 1917, respectively) accounted for 66% of the total reads, and 37% of the total reads belonged to *Psychoda phalaenoides*. In addition to insect sequences, we also found eDNA from other arthropods such as mites and springtails, as well as one species of parasitic nematode *Bunostomum phlebotomum* Railliet, 1900, and not surprisingly, cow (*Bos taurus*). We did not include a mock sample in this study, as several previous studies have shown accordance between tissue‐extracted DNA amounts added to the mock and subsequent DNA reads (Sigsgaard et al., 2019; Thomsen et al., 2016), and since we do not make any strong assumptions regarding quantitative estimates such as number of individuals or biomass in this study.

### 3.3 Taxonomic and functional diversity

Our study detected eDNA from species across both taxonomic and functional groups of invertebrates (Table 1, Figure 3). Specifically, we detected eDNA from several species representing the following groups: (i) species feeding entirely or mainly on the dung itself (e.g., Scarabaeidae, Hydrophilidae, Staphylinidae, Sepsidae, Sphaeroceridae, Scathophagidae); (ii) predatory species living as facultative or obligate carnivores feeding on other invertebrates in the dung (e.g., Histeridae, Muscidae); (iii) species feeding on the fungi associated with the dung (e.g., Collembola, Acari); (iv) species using the dung as habitat where they can e.g. hide in moist crevices during the day (Dermaptera); (v) external or internal parasites of the cow (Bovicoliidae and Ancylostomatidae, respectively); and (vi) species that have no association with the dung, but are present in the grassland habitat and thus in the near surroundings (e.g., Curculionidae, Phalacridae, Aphididae, Miridae, Pentatomidae, Formicidae).

### 3.4 Differentiation of cow dung assemblages by habitat

**FIGURE 4:**
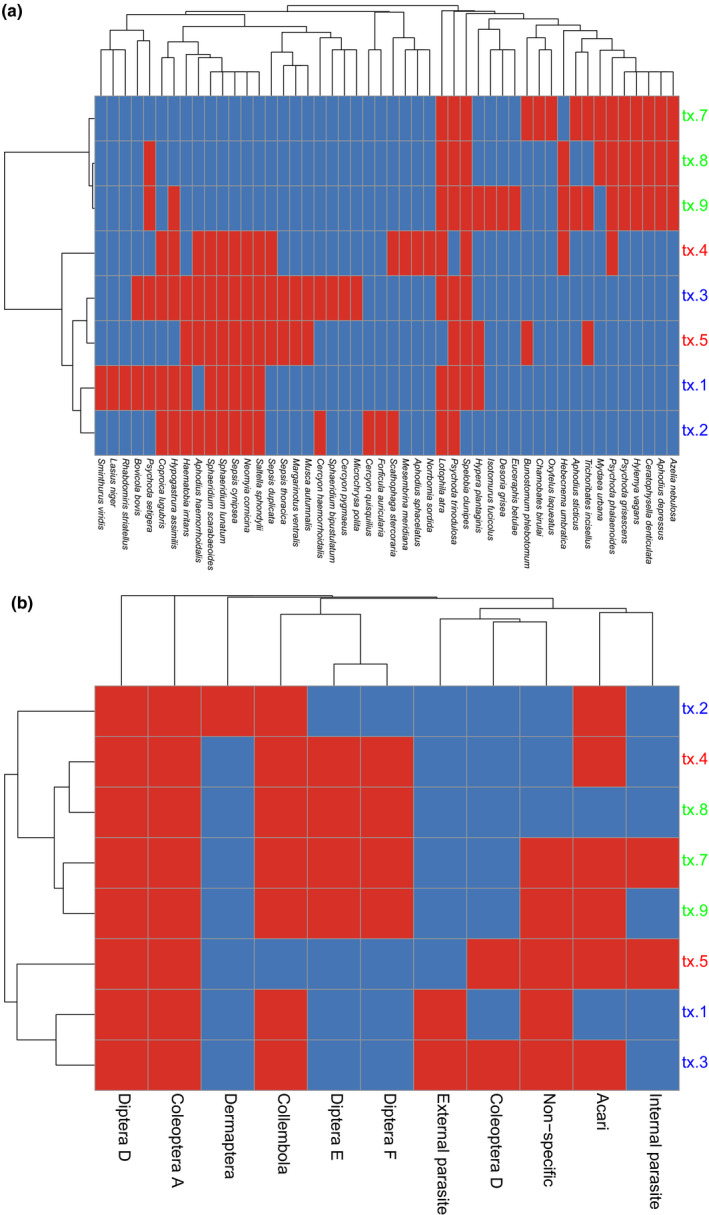
Cluster analyses and heat map at the level of (a) species and (b) trophic group (based on Skidmore, 1991), showing presence (red) and absence (blue) of each species or trophic group found in the dung samples from dry grassland (tx.1–tx.3), meadow (tx.4–tx.5), and forest (tx.7–tx.9)

**FIGURE 5:**
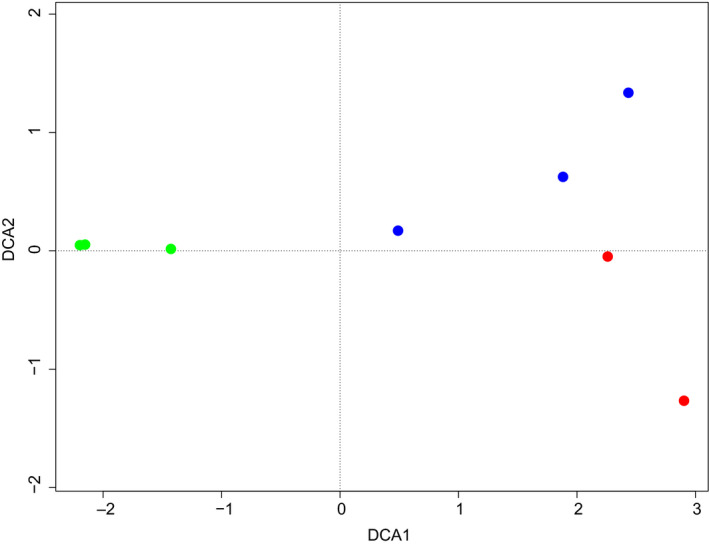
Detrended correspondence analysis at species level showing samples from three habitats; meadow (red circles), dry grassland (blue circles) and forest (green circles)

**FIGURE 6:**
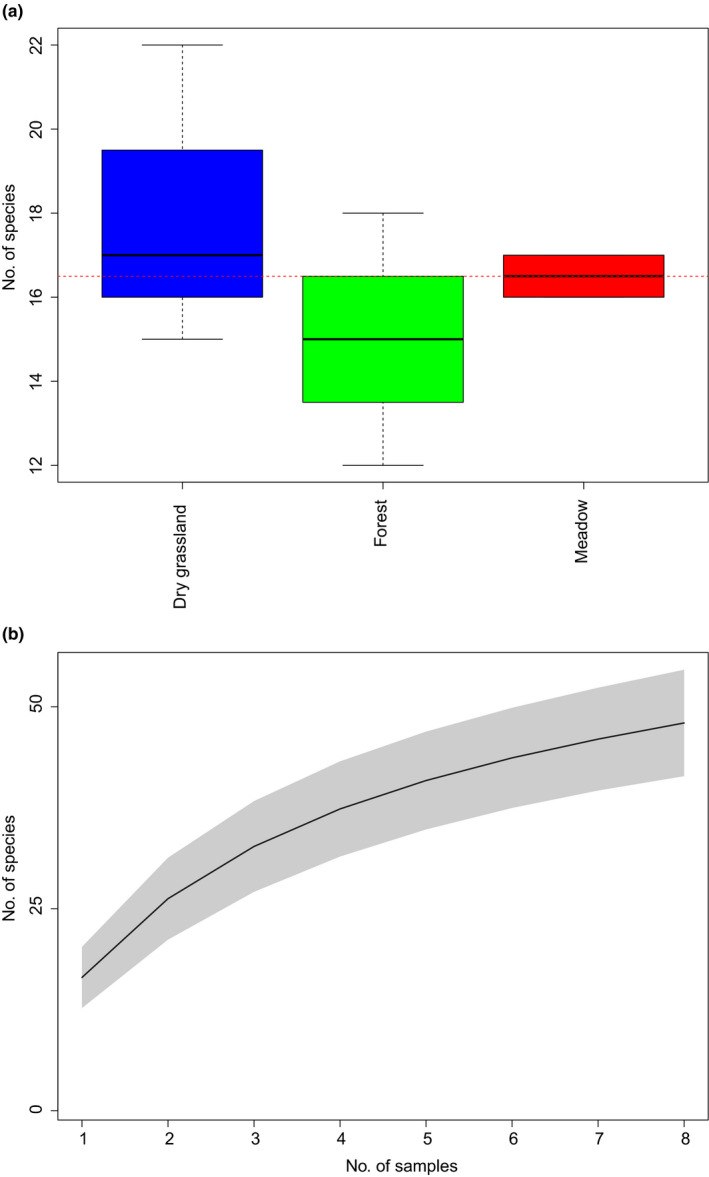
Species richness in cow dung samples shown for (a) each habitat; and (b) as an accumulation curve for the entire study area (grey shading, 95% CIs based on unconditional *SD*)

The PERMANOVA test indicated that 72% (*p* = .02) of the variation in similarity between sampling sites could be explained by habitat type. Results from the cluster analysis (heat map) showed that the cow dung assemblages obtained from the eDNA segregate into forest and open grassland (meadow and dry grassland), and that this signal is driven by several taxa that only occur in the forest dung samples (Figure 4a). No species occurred in all eight dung samples. In contrast, each trophic group occurred in at least two different habitats, with the exception of Dermaptera and external parasites, which were only detected in the dry grassland habitat (Figure 4b). However, both these groups were represented by a single species. Correspondence analyses also indicated that samples from forest formed a distinct group (Table 1, Figure 5). The forest habitat also had the lowest number of associated invertebrate species (Figure 6a), although there was no statistically significant difference in species richness between habitat types (ANOVA, *p* = .5).

### 3.5 Sequencing depth and replication

Rarefaction curves indicated that sequencing depth was sufficient (Figure S2), but accumulation curves indicate that greater sampling effort would increase detected diversity (Figure 6b, Figure S3).

## 4 DISCUSSION

Wild herbivores in natural population densities are associated with large quantities of dung, which support a rich and specialized community of invertebrates and fungi (Byk & Piętka, 2018; Richardson, 2001). However, wild megafauna have undergone extinction (Sandom et al., 2014) or experienced dramatic decline (Dirzo et al., 2014; Ripple et al., 2015) all over the world. Trophic rewilding supports the existence of dung communities, though population regulation based on resource availability and animal condition as in the Rewilding Mols area is rare for large herbivores in a European context. Discontinuity of grazing, abandonment and habitat modification thus continue to pose threats to the fauna associated with herbivore dung (Carpaneto et al., 2007; Nichols et al., 2009; Tonelli et al., 2018). In order to investigate the effect of rewilding practices on general biodiversity, extensive monitoring is needed. However, species‐rich groups such as arthropods can be very resource demanding to monitor, and alternative noninvasive genetic approaches for studying dung fauna are appealing.

In this study, we explore the potential of eDNA metabarcoding as a supplementary approach to obtain information on species compositions and associations in complex dung assemblages. We demonstrate that samples of cow dung can be a valuable source of eDNA from terrestrial invertebrates—particularly insects—associated with the dung. We found eDNA from a range of species representing both taxonomic and functional diversity, including herbivores (e.g., dung beetles, dung flies), predators (e.g., clown beetles), fungal feeders (e.g., springtails, mites) and parasites (e.g., lice, nematodes). Several of these groups, such as the dung beetles, are completely dependent on dung, and are therefore especially relevant in the context of rewilding. Additionally, eDNA was obtained from a few common grassland species not associated with dung, which probably represented random contact with the dung (e.g., ants, shield bugs, weevils). We also found that the cow dung assemblages obtained from eDNA were differentiated among habitats with forest being different from open grassland (meadow and dry grassland). Finally, accumulation curves show that our approach was not exhaustive, indicating that more comprehensive dung fauna analyses can be made using an eDNA approach with more cowpat samples per sampling site. Importantly, this final point also illustrates that our study should be regarded as proof‐of‐concept of the approach, given the limited number of samples and spatial replication. Nonetheless, it can hopefully show the way for more extensive studies on dung fauna ecology using eDNA.

### 4.1 Differentiated cow dung assemblages obtained from eDNA

The dung assemblages recovered from eDNA separated according to openness of the habitat, in that forest was clearly distinct from open grassland habitats. This indicates that temperature and light could be important factors for defining the dung assemblages in this particular locality, as these are probably the abiotic factors differing most between the sampled habitats. It is noteworthy that forest dung assemblages were differentiated from the assemblages of the open habitats despite the fact that forest and meadow are the habitats situated most closely together geographically, while the dry grassland habitat is further away (Figure 1). The forest habitat had the lowest richness of associated invertebrates (Figure 6a), which is in accordance with the temperature‐dependence of many dung‐associated insects (Landin, 1961). Nevertheless, the forest habitat seems to have a distinct assemblage, and several species were only obtained from forest dung samples (Figure 4a). Interestingly, the dung beetles *Aphodius sticticus* Panzer, 1798, and *Aphodius depressus* Kugelann, 1792, were only found in forest samples. Both of them, and especially *Aphodius sticticus*, is associated with forest habitats and lower temperatures compared to the two other dung beetle species *Aphodius haemorrhoidalis* Linnaeus, 1758, and *Aphodius sphacelatus* Panzer, 1798, found in this study (Roslin et al., 2014), which were only found in open habitats. Also, the springtail *Ceratophysella denticulata* Bagnall, 1941, has been described as usually occurring in humid conditions (Fjellberg, 1998), and this species was also only obtained in forest habitat (in all three samples). Focusing on the functional groups for habitat differentiation, it seems that the species mainly responsible for the separate clustering of the forest habitat are facultative and obligate carnivorous dipterans (Diptera E and F), while herbivorous dung‐feeding dipterans (Diptera D) and dung‐feeding beetles (Coleoptera A) are found across all samples and habitats (Figure 4b). It should be noted that these findings do not reflect the actual number of taxa in the dung samples, as PCR replication was probably insufficient.

### 4.2 Other sources of invertebrate eDNA in cow dung

Some of the invertebrate DNA obtained from dung in this study might not be eDNA in the strict sense, but could originate from for instance eggs, larvae, or small imagoes. Such individuals may easily have been overlooked during sampling and DNA extraction. However, such detections would still indicate presence of the species in question. In contrast, larger more mobile individuals might potentially carry eDNA from other invertebrate species on their surface and thus “contaminate” dung samples with exogenous eDNA. Dung‐associated species may also carry dung from previous visits to other cowpats. Such transport of eDNA potentially leading to false positive results has been a recurring concern in eDNA studies (Goldberg et al., 2016; Thomsen & Willerslev, 2015). However, most studies indicate that eDNA composition reflects local species composition at a fine spatial scale (Port et al., 2016; Tillotson et al., 2018; Yoccoz et al., 2012), and we assume that such contamination is very infrequent compared to the amounts of eDNA deposited by species in direct contact with the dung.

### 4.3 Caveats of eDNA metabarcoding studies

Besides the above‐mentioned challenges of establishing how and why invertebrate eDNA can be found in samples of cow dung, other issues should also be carefully considered in eDNA metabarcoding, and we discuss the most important ones in the following. Although the primers used in this study were designed for metabarcoding of diverse invertebrates and were successfully tested both in silico and in vitro, a few invertebrate groups (e.g., Hirudinea or leeches) are less compatible or incompatible with these primers (Elbrecht & Leese, 2017). Also, in vivo the primers appeared to amplify quite a large proportion of nontarget sequences (less than half of the reads in the present study were from metazoans), a general metabarcoding issue which has been highlighted in previous studies (Alberdi et al., 2018). Thus, some invertebrate groups may have been amplified inefficiently or not at all in our study. The resulting metabarcode provided high taxonomic resolution however, with only nine taxa (16%) that could not be identified to species. This resolution was also a result of the availability of a well‐curated and (at least in our case) comprehensive database of reference sequences, namely the BOLD database. The existence of erroneous sequences such as sequences that are wrongly identified taxonomically are a significant issue in large public databases such as GenBank (Steinegger & Salzberg, 2020) and can lead to false positive or negative results, and lower taxonomic resolution. False positive or negative results can also arise from sequencing or PCR errors, as well as from contamination from various sources (Thomsen & Willerslev, 2015). In this study, to avoid false positive results we applied software for removing sequencing reads likely to be the result of sequencing or PCR errors, and also required sequences to be present in two PCR replicates to be retained in the final data. This approach could be further improved by running a larger number of PCR replicates, something which our accumulation analyses indicate would also provide a more exhaustive coverage of the taxonomic diversity in the samples. One of our samples failed to produce visible amplification, perhaps because of PCR inhibition despite the use of BSA. As both plants and faecal samples can contain a variety of substances inhibitory to PCR (Schrader et al., 2012), some level of inhibition is to be expected in dung samples, but several measures can be taken to reduce it if needed (Schrader et al., 2012). While we did not experience issues with contamination in the current study, it is important to be aware of potential contamination throughout the eDNA workflow by, for instance, using a separate laboratory dedicated to extraction of eDNA samples. A general issue with eDNA data, especially that resulting from PCR amplification, is that the ability to make quantitiative inferences is less straight‐forward than for traditional monitoring approaches (Taberlet et al., 2018). However, several studies conducted in the field have indicated that at least for aquatic vertebrates, there appears to be a correlation between biomass and/or number of individuals, and eDNA concentration or sequencing read numbers (Biggs et al., 2015; Thomsen et al., 2016; Yamamoto et al., 2016). An important factor in eDNA studies is the degradation time of DNA which can range from hundreds of thousands of years in ancient permafrost (Willerslev et al., 2003) to a few days or weeks in contemporary water samples (Thomsen, Kielgast, Iversen, Møller, et al., 2012; Thomsen, Kielgast, Iversen, Wiuf, et al., 2012). The eDNA persistence in dung remains unknown and should be a focus of future studies. In temperate soil, DNA can potentially be obtained many years after deposition from the organisms (Yoccoz et al., 2012), but as dungpats are produced continuously, it should be possible to avoid sampling “old” eDNA.

### 4.4 Future perspectives

Environmental DNA metabarcoding of dung has perspectives for both fundamental and applied research, as well as for monitoring and conservation of dung assemblages. The approach could improve estimates of species composition, abundances and distributions by supplementing existing methods, and allow for more extensive long‐term monitoring of such variables (Hallmann et al., 2017). In the case of endangered species, the high sensitivity and noninvasiveness of the eDNA approach makes the method especially advantageous. However, as the metabarcoding approach makes it possible to study a wide diversity of species simultaneously, these species can be included in a broadly targeted approach, which also includes less prolific species that may nonetheless be vital to the ecosystem. In this way, unknown species or unknown ecological interactions may also be detected. As an interesting example from this study, we found eDNA from the moth fly species *Psychoda grisescens* Tonnoir, 1922 (Psychodidae). This species is not yet recognised as a Danish species (Petersen & Meier, 2001), but since the faunistics of moth flies is poorly known, it might well occur in Denmark unnoticed or not yet registered. Indeed, specimens seem to have been collected from Denmark in a previous study (Espíndola & Alvarez, 2011), and since all nine Danish species of the genus (www.allearter‐databasen.dk, accessed 14 September 2020) have sequences deposited in BOLD, incomplete database coverage cannot explain the detection. This case illustrates the usefulness of the present eDNA approach for obtaining information on unknown species. Importantly, we stress that issues related to unknown factors such as eDNA quantification, degradation and transport should be studied further in dung samples before the approach can be considered for integration into monitoring. Finally, as we are well aware of the limited number of samples in our study, we recommend that the current approach of detecting dung‐associated fauna using eDNA metabarcoding is repeated in other settings.

## AUTHOR CONTRIBUTIONS

E.E.S., K.O., M.D.D.H., O.L.P.H., T.T.H., J.‐C.S., and P.F.T. were responsible for the conceptualization and design of the study. E.E.S., K.O., and P.F.T. were responsible for data acquisition. E.E.S., and P.F.T. were responsible for data analysis. E.E.S., K.O., M.D.D.H., O.L.P.H., T.T.H., J.‐C.S., and P.F.T. were responsible for writing the manuscript.

## Supporting information

Figures S1‐S3

## Data Availability

Illumina raw sequence data and the final ASV sequences and ASV table are available from the Dryad Digital Repository at https://doi.org/10.5061/dryad.f7m0cfxtp (Sigsgaard, Olsen, et al., 2020).
