# Supplementary material for: Environmental DNA metabarcoding of cow dung reveals taxonomic and functional diversity of invertebrate assemblages
Source: Mol Ecol. 2020 Dec 15;30(13):3374–89. doi: 10.1111/mec.15734 (PMC8359373; doi:10.1111/mec.15734)
Supplement: Supplementary file 1 — Figures S1‐S3 [file MEC-30-3374-s001.docx]

**Supplemental Information for:**

**Environmental DNA metabarcoding of cow dung reveals taxonomic and functional diversity of invertebrate assemblages**

Eva Egelyng Sigsgaard, Kent Olsen, Morten D. D. Hansen, Oskar Liset Pryds Hansen,

Toke Thomas Høye, Jens-Christian Svenning, Philip Francis Thomsen


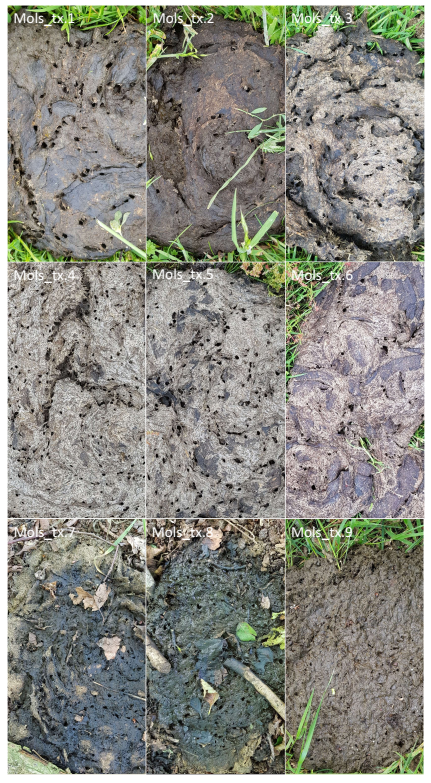


Figure S1. Photo plate of the 9 dung samples. Sample tx.6 did not produce any PCR amplification. Samples were collected in 2019 on the following dates: tx.1-tx.3: June 12, tx.4-tx.6: June 14, and tx.7-tx.9: June 17. Photos: Kent Olsen


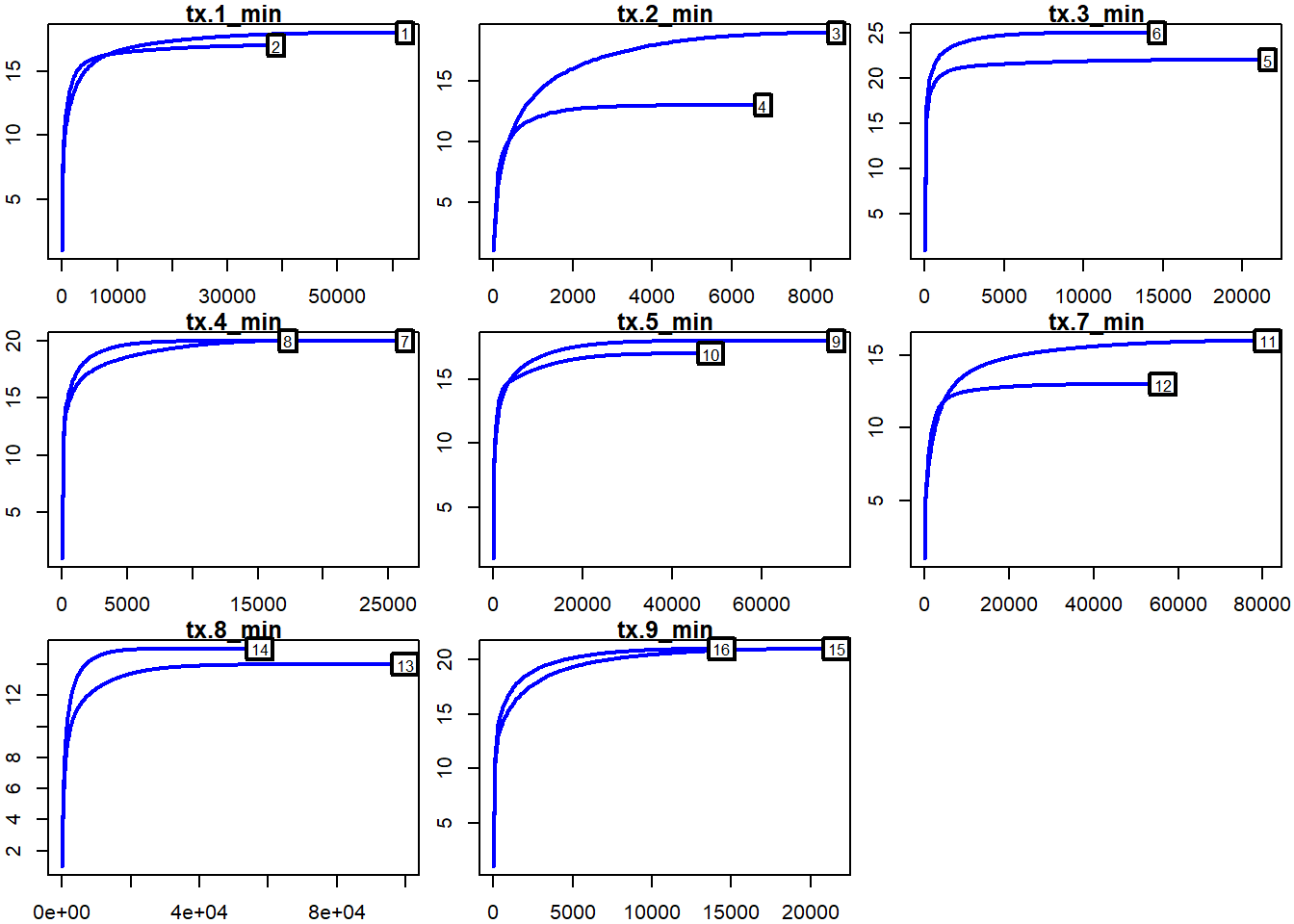


Figure S2. Rarefaction curves for PCR replicates


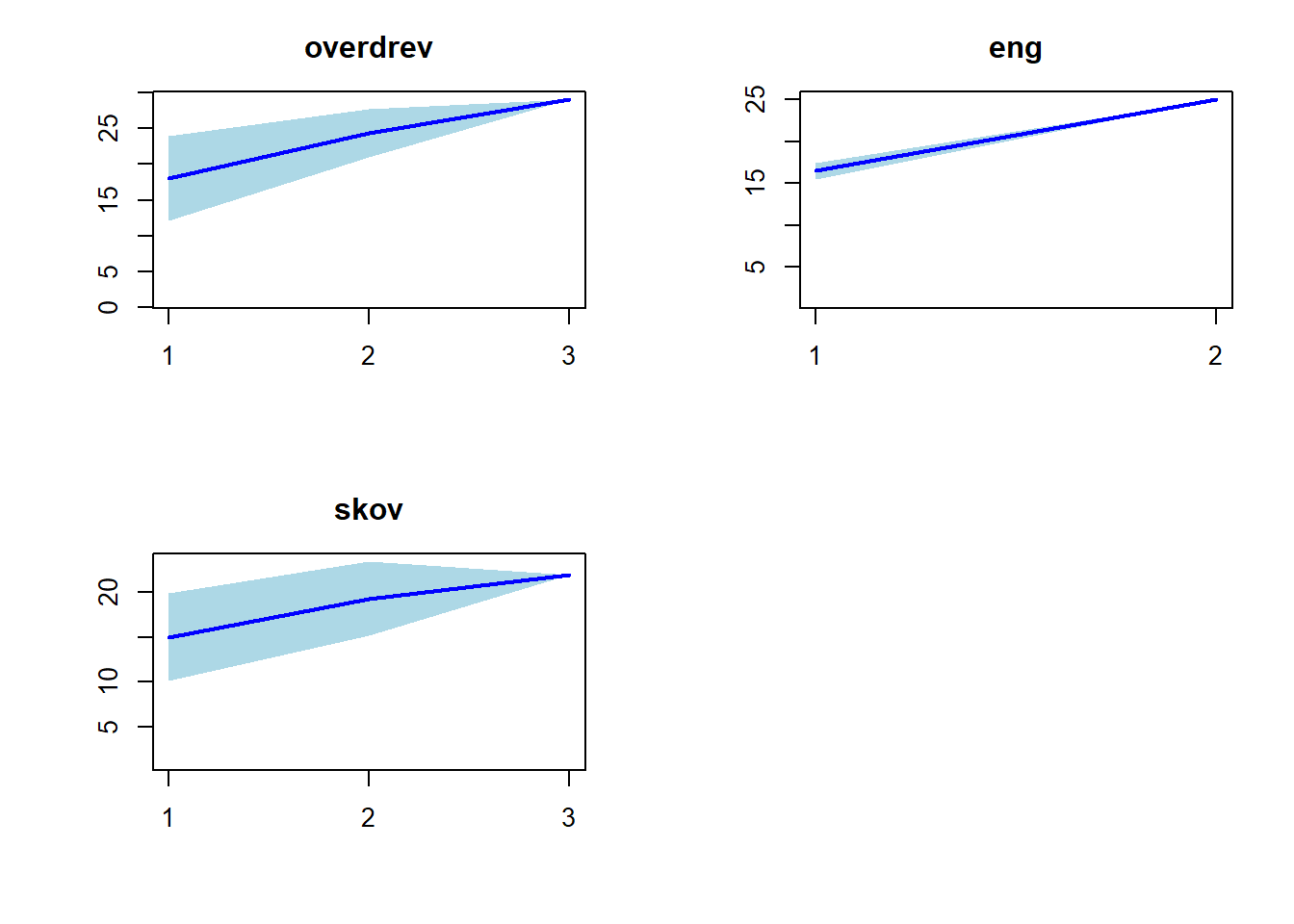


Figure S3. Accumulation curves of species richness for the cow dung samples from each habitat.
